# Mild therapeutic hypothermia is superior to controlled normothermia for the maintenance of blood pressure and cerebral oxygenation, prevention of organ damage and suppression of oxidative stress after cardiac arrest in a porcine model

**DOI:** 10.1186/1479-5876-11-124

**Published:** 2013-05-20

**Authors:** Petr Ostadal, Mikulas Mlcek, Andreas Kruger, Svatava Horakova, Marcela Skabradova, Frantisek Holy, Tomas Svoboda, Jan Belohlavek, Vladimir Hrachovina, Ludek Taborsky, Vlasta Dudkova, Hana Psotova, Otomar Kittnar, Petr Neuzil

**Affiliations:** 1 Cardiovascular Center, Na Homolce Hospital, Prague 15030, Czech Republic; 2 Department of Physiology, First Faculty of Medicine, Charles University in Prague, Prague 12000, Czech Republic; 3 2nd Department of Medicine - Department of Cardiovascular Medicine, First Faculty of Medicine, General University Hospital and Charles University in Prague, Prague 12000, Czech Republic; 4 Department of Clinical Biochemistry, Hematology, and Immunology, Na Homolce Hospital, Prague 15030, Czech Republic; 5 Department of Nuclear Medicine, Na Homolce Hospital, Prague, 15030, Czech Republic

**Keywords:** Cardiac arrest, Mild hypothermia, Normothermia, Post-cardiac arrest syndrome, Extracorporeal membrane oxygenation, Cerebral oxygenation, Blood pressure, Organ protection, Oxidative stress

## Abstract

**Background:**

Mild therapeutic hypothermia (HT) has been implemented in the management of post cardiac arrest (CA) syndrome after the publication of clinical trials comparing HT with common practice (ie, usually hyperthermia). Current evidence on the comparison between therapeutic HT and controlled normothermia (NT) in CA survivors, however, remains insufficient.

**Methods:**

Eight female swine (*sus scrofa domestica*; body weight 45 kg) were randomly assigned to receive either mild therapeutic HT or controlled NT, with four animals per group. Veno-arterial extracorporeal membrane oxygenation (ECMO) was established and at minimal ECMO flow (0.5 L/min) ventricular fibrillation was induced by rapid ventricular pacing. After 20 min of CA, circulation was restored by increasing the ECMO flow to 4.5 L/min; 90 min of reperfusion followed. Target core temperatures (HT: 33°C; NT: 36.8°C) were maintained using the heat exchanger on the oxygenator. Invasive blood pressure was measured in the aortic arch, and cerebral oxygenation was assessed using near-infrared spectroscopy. After 60 min of reperfusion, up to three defibrillation attempts were performed. After 90 min of reperfusion, blood samples were drawn for the measurement of troponin I (TnI), myoglobin (MGB), creatine-phosphokinase (CPK), alanin-aminotransferase (ALT), neuron-specific enolase (NSE) and cystatin C (CysC) levels. Reactive oxygen metabolite (ROM) levels and biological antioxidant potential (BAP) were also measured.

**Results:**

Significantly higher blood pressure and cerebral oxygenation values were observed in the HT group (P<0.05). Sinus rhythm was restored in all of the HT animals and in one from the NT group. The levels of TnI, MGB, CPK, ALT, and ROM were significantly lower in the HT group (P<0.05); levels of NSE, CysC, and BAP were comparable in both groups.

**Conclusions:**

Our results from animal model of cardiac arrest indicate that HT may be superior to NT for the maintenance of blood pressure, cerebral oxygenation, organ protection and oxidative stress suppression following CA.

## Background

Mild therapeutic hypothermia (HT) was introduced into the clinical management of cardiac arrest survivors after the publication of the results of two clinical trials showing a benefit compared with standard treatment [1,2], and has been included in the guidelines for post-cardiac arrest care from the American Heart Association [3] and the European Resuscitation Council [4]. The implementation of HT into clinical practice was also supported by meta-analyses of these clinical trials [5,6]. Recently, however, several authors presented a critical view of the current evidence for the protective role of HT in post-cardiac arrest syndrome. In analyses of the available randomized data, they showed that the evidence for the benefits of HT in cardiac arrest survivors remains inconclusive [7,8]. Their criticisms were based on the small size of published trials, low-quality data and non-negligible risks of systematic and random errors [7,8]. Furthermore, in the published studies, HT was compared with standard care without specific temperature management, ie, usually with spontaneously developed fever. The deleterious effects of fever and hyperthermia have been repeatedly observed in a wide population of critically ill patients [9] as well as in cardiac arrest survivors [10,11]. The relative lack of evidence regarding the benefits of mild therapeutic HT in cardiac arrest survivors has led to the establishment of a large multicenter randomized controlled trial comparing mild therapeutic HT with controlled normothermia (NT) in CA survivors (the Target Temperature Management trial), which is currently underway [12].

Presently, there is minimal research comparing mild therapeutic HT with controlled NT in either human studies [13] or animal experiments [14]. Therefore, the aim of the present study was to compare the effects of mild therapeutic HT and controlled NT on several clinically relevant outcomes in an animal model of cardiac arrest. We tested the hypothesis that mild HT is superior to controlled NT in the maintenance of blood pressure and cerebral oxygenation, resuscitability, prevention of organ damage and oxidative stress suppression after cardiac arrest in a porcine model.

## Methods

The present study was approved by the Charles University 1^st^ Medical School Institutional Animal Care and Use Committee and performed at the Animal Laboratory, Department of Physiology, 1^st^ Medical School, Charles University in Prague, Czech Republic, in accordance with Act No 246/1992 Coll. regarding the protection of animals against cruelty.

### Animal model

Eight female swine (*Sus scrofa domestica*, Landrace × Large white crossbreed), four to five months of age, with a mean body weight of 45 kg were included in the experiment. After 24 hr of fasting, general anesthesia was induced by administration of azaperone (2 mg/kg IM) followed by atropine sulphate (0.02 mg/kg IM) and ketamine hydrochloride (15 to 20 mg/kg IM). Anesthesia was continued with initial propofol and morphine boluses (2 mg/kg IV and 0.1 to 0.2 mg/kg IV, respectively) and animals were orotracheally intubated. Continuous IV infusions of propofol (8 to 10 mg/kg/hr) and morphine (0.1 to 0.2 mg/kg/hr) were used to maintain anesthesia. The doses were adjusted according to physiological parameters, photoreaction, corneal and palpebral reflexes, lacrimation and spontaneous movement. At the end of the experiment, potassium chloride (2 meq/kg) in conjunction with general anesthesia was used to euthanize animals.

Bilateral femoral (arterial and venous) and jugular approaches were used for multiple sheath insertions by the standard percutaneous Seldinger technique. An initial rapid IV infusion of 1000 ml of normal saline was given after anesthesia induction, followed by a continuous IV drip of 200 to 500 ml/hour to reach and maintain a mean right atrial pressure of 3 to 7 mmHg. An unfractionated heparin bolus (100 U/kg IV) was given after sheath placement, followed by a continuous IV infusion of 50 U/kg/h to maintain an activated clotting time of 180 to 250 s. Values were monitored every hour with the Hemochron Junior+ Microcoagulation System (ITC, USA).

Ventilation was provided by a Hamilton G5 ventilator (Hamilton Medical AG, Switzerland) set to INTELLiVENT - Adaptive Support Ventilation (ASV) mode. The ventilator was set to maintain an SpO_2_ of 95% to 99%, and an EtCO_2_ of 4.5 to 5.6 kPa. During the non-treated phase of cardiac arrest, the animals were not ventilated. During the ECMO-treated phase of cardiac arrest, minute ventilation was set and maintained at 100% of the predicted minute volume based on ideal body weight. The fraction of inspired oxygen was set to 21%.

Ventricular fibrillation was induced by a high-frequency burst from a diagnostic decapolar catheter (Response CSL, St. Jude Medical, USA) advanced to the right ventricular apex through a jugular venous approach. The stimulation current amplitude was amplified to two times the pacing threshold.

### Vital functions monitoring

Arterial pressure was measured using routine invasive pressure transducers (Truwave, Edwards Lifesciences, LLC, USA) through a pigtail catheter inserted into the aortic arch. A Swan-Ganz catheter was introduced via a femoral vein to the pulmonary artery. Electrocardiogram, invasive blood pressures (aortic arch and jugular vein), pulse oximetry, capnometry, core temperature (pulmonary artery) and invasive central venous oxygen saturation were continuously monitored in all animals (Monitor Life Scope TR, Nihon Kohden, Japan and Vigilance II, Edwards Lifesciences, USA). Brain oxygenation levels were measured using near-infrared spectroscopy (INVOS Cerebral/Somatic Oximeter, Somanetics, USA).

### ECMO

The ECMO circuit consisted of a Levitronix Centrimag console (Thoratec, USA), centrifugal pump, and tubing set with HMO 70000 Adult Microporous Membrane Oxygenator with Softline Coating (MAQUET Cardiopulmonary AG, Germany) and a mechanical gas blender (Sechrist, USA). Biomedicus cannulae (Medtronic, USA) were introduced percutaneously using the standard Seldinger technique after repeated dilations of the femoral artery and vein. The venous inflow cannula (21F) was inserted into the inferior vena cava slightly below the right atrium (the tip position was checked by fluoroscopy) and the femoral arterial outflow cannula (15F) was inserted into the femoral artery. Body temperature was regulated and kept constant by the oxygenator heater unit. Blood gases were monitored continuously in the blood leaving the oxygenator (CDI™ Blood Parameter Monitoring System 500, Terumo Cardiovascular Systems Corporation, USA). During the non-treated phase of cardiac arrest, the pump flow rate was set to 0.5 L/min and oxygen/air flow was stopped. During the ECMO-treated phase following cardiac arrest, the pump flow rate was set to 4.5 L/min and oxygen/air flow was repeatedly adjusted to maintain pO_2_ and pCO_2_ in the ranges of 10 to 15 kPa and 4.0 to 6.5 kPa, respectively, in blood leaving the oxygenator. The circuit, console function, and initial settings were controlled and directed by a perfusionist.

### Experimental protocol

**Figure 1:**
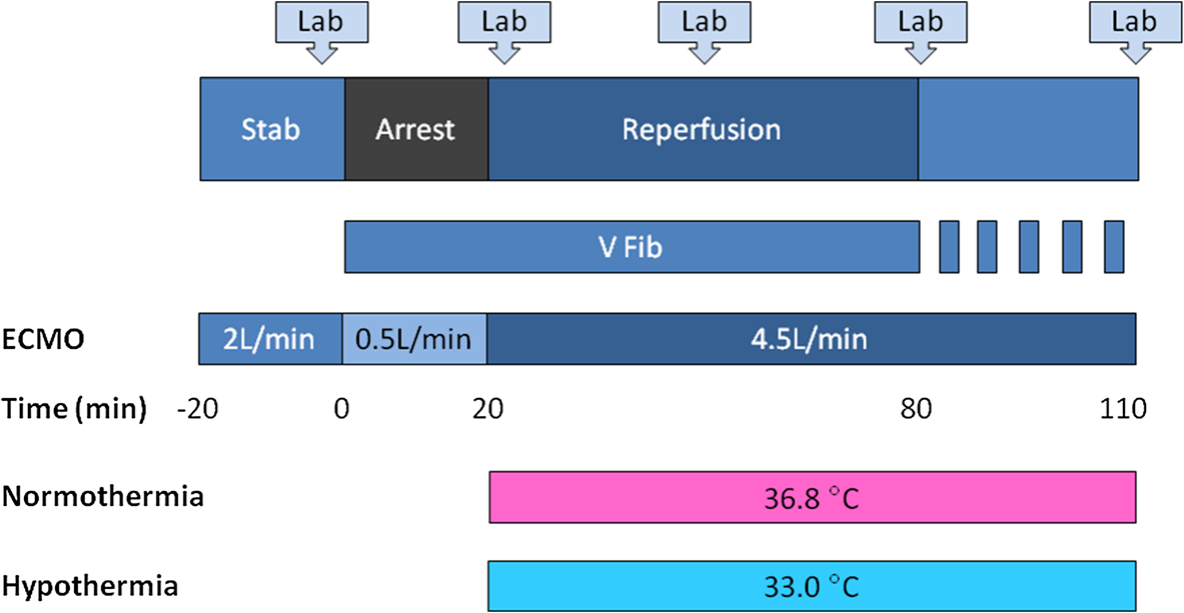
**Study protocol.** Veno-arterial extracorporeal membrane oxygenation (ECMO) was established and at minimal ECMO flow (0.5 L/min) ventricular fibrillation (V Fib) was induced by rapid ventricular pacing. After 20 min of cardiac arrest, circulation was restored by increasing the ECMO flow to 4.5 L/min (100 mL/kg/min); 90 min of reperfusion followed. After 60 min of reperfusion, up to three defibrillation attempts were performed. Blood samples for the measurement of laboratory parameters (Lab) were taken before and 1 min after arrest and then at 30, 60, and 90 min of reperfusion. Stab, stabilization.

After insertion of all catheters and establishment of ECMO the animals were stabilized for 10 min. Cardiac arrest was induced and maintained for 20 min before circulation was restored by increasing the ECMO flow rate to 4.5 L/min (100 mL/kg/min). After 60 min of reperfusion, defibrillation was attempted up to three times (300 – 360 – 360 J) to restore sinus rhythm, and the animals were followed for an additional 30 min. The ECMO support continued the whole 90 min of reperfusion irrespective of defibrillation results (Figure 1).

Animals were randomly assigned to one of the two groups: NT or HT, with four animals per group. The core temperature of the animals in the NT group was maintained at 36.8°C throughout the experiment. The animals in the HT group were cooled to 33.0°C at the beginning of reperfusion; the target temperature was attained in 5 min in all HT animals and was maintained until the end of experiment.

To simulate clinical conditions, norepinephrine was administered when the mean arterial blood pressure remained below 60 mmHg at 10 min after the restoration of circulation. The norepinephrine IV infusion was started at 0.2 μg/kg/min; this dose could be doubled or decreased by one-half at 15 min, 20 min, and then every 10 min until the end of the experiment, with a maximal dose of 1.6 μg/kg/min.

### Laboratory tests

Blood samples were drawn after 90 min of reperfusion and were immediately centrifuged. Serum was stored at −70°C until levels of troponin I (TnI), myoglobin (MGB), creatine phosphokinase (CPK), alanine aminotransferase (ALT), neuron-specific enolase (NSE), and cystatin C (CysC) were measured. Blood samples for the measurement of reactive oxygen metabolites (ROM) and biological antioxidant potential (BAP) were drawn before CA, 1 min after restoration of blood flow, and after 30, 60 and 90 min of reperfusion. ROM and BAP tests were performed immediately after samples were obtained.

TnI and MGB levels were measured using a chemiluminescent immunoassay (Access Immunoassay System, Beckman Coulter Inc, USA). CPK and ALT levels were measured according to the method outlined by the International Federation of Clinical Chemistry, using the Synchron System (Beckman Coulter Inc, USA). NSE was measured with an immunoradiometric assay kit (Beckman Coulter, USA). CysC was measured with a Cystatin C immunoassay kit (Gentian AS, Norway) utilizing the immunoturbidimetric method. ROM and BAP were measured with the Fras 4 Evolvo free radical analytical system (H&D srl, Italy).

### Statistical analysis

Results are expressed as median (min; max). Mann Whitney test was used to assess differences between treatment groups in mean arterial blood pressure, norepinephrine dose, brain oxygen saturation, levels of oxidative stress markers (ROM and BAP) and all other laboratory parameters. Fisher’s exact test was used to test for a difference in resuscitability. Differences were considered to be statistically significant at P<0.05. All statistical analyses were performed using GraphPad Prism 5.0 software (GraphPad, USA).

## Results

### Blood pressure, brain oxygen saturation, resuscitability

**Figure 2:**
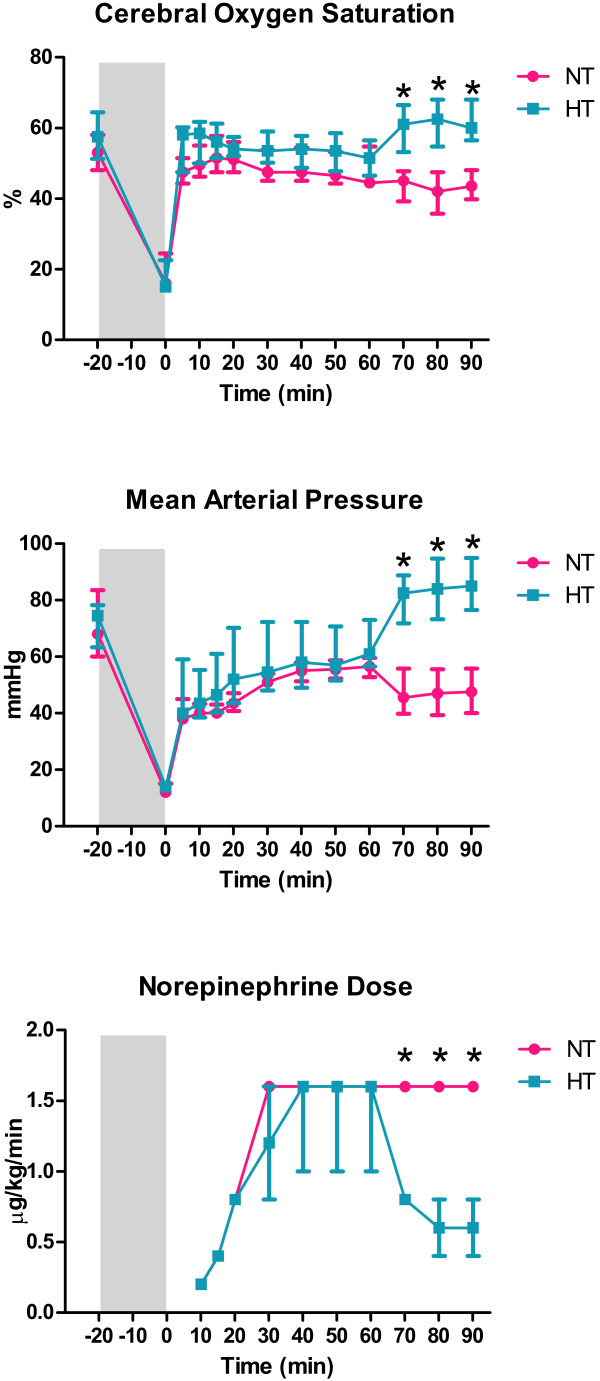
**Brain oxygen saturation, mean arterial pressure and norepinephrine dose progression over the course of the experiment.** Values are expressed as median (min; max). *P<0.05; NT normothermia; HT hypothermia.

The mean arterial blood pressures, brain oxygen saturations and norepinephrine doses were not significantly different between treatment groups during the first 60 min of reperfusion (Figure 2). At 70, 80, and 90 min, the mean arterial blood pressures and brain oxygen saturations were significantly higher in the HT group, despite the fact that the norepinephrine doses administered to this group were significantly lower compared with the NT group (P<0.05; Figure 2). Defibrillation at 60 min was successful in all HT animals and in one of the NT animals after three attempts.

### Organ damage

**Figure 3:**
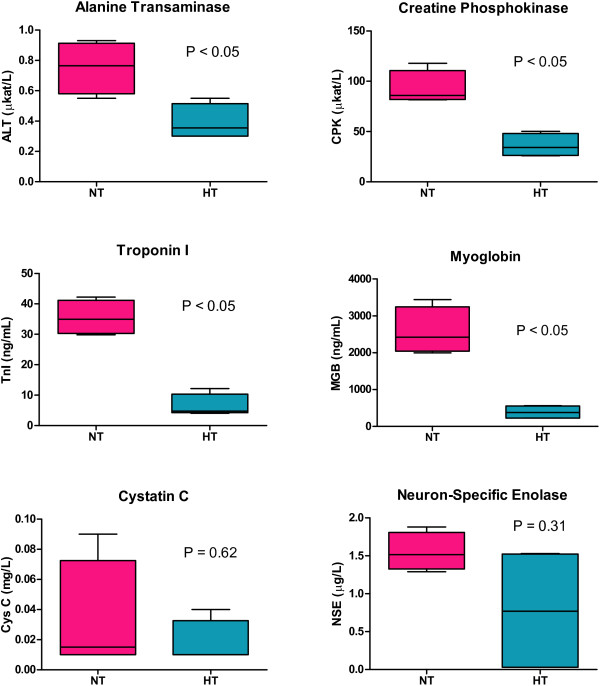
**Serum levels of biomarkers of organ injury.** Values are expressed as median (min; max). NT, normothermia; HT hypothermia.

Laboratory parameters were analyzed in blood samples obtained at 90 min of reperfusion. Levels of ALT, CPK, MGB, and TnI were significantly lower in the HT group compared with NT group (ALT: 0.36 (0.30; 0.55) versus 0.79 (0.65; 1.27) μkat/L, P<0.05; CPK: 34.2 (26.0; 50.29) versus 86.0 (81.6; 117.8) μkat/L, P<0.05; MGB: 379.8 (225.3; 566.0) versus 2423.0 (1995.0; 3442.0) ng/L, P<0.05; TnI: 4.8 (4.0; 12.2) versus 35.0 (29.8; 42.2) ng/L, P<0.05) (Figure 3). The levels of both Cys C and NSE were close to the lower limit of detection, and no significant differences between the HT and NT groups were observed (Cys C: 0.01 (0.01; 0.04) versus 0.02 (0.1; 0.9) ng/L, P=0.62; NSE: 0.77 (0.03; 1.53) versus 1.52 (1.29; 1.88) ng/L, P=0.31) (Figure 3).

### Oxidative stress

**Figure 4:**
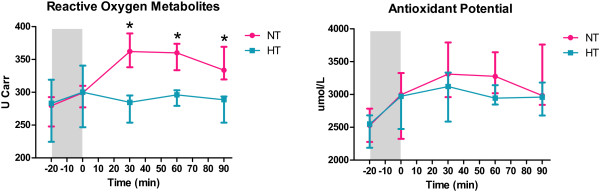
**Serum levels of oxidative stress parameters.** Values are expressed as median (min; max). NT, normothermia; HT hypothermia.

The levels of ROM prior to and immediately after cardiac arrest were comparable between the HT and NT groups, but were significantly lower in the HT group in samples obtained at 30, 60 and 90 min of reperfusion (Figure 4). The levels of BAP were comparable between the HT and NT groups in all measurements.

## Discussion

The present study found that mild therapeutic HT (33°C) is superior to controlled NT (36.8°C) for the maintenance of blood pressure and brain oxygen saturation, protection from organ damage and suppression of oxidative stress following cardiac arrest.

The use of mild therapeutic HT in the treatment of cardiac arrest survivors has recently been questioned due to a low level of evidence of its benefits [7,8]; in addition, spontaneous hyperthermia that may have occurred in the control patients in published clinical trials seems to be a significant risk factor [10,11]. However, to date, there have been few publications directly comparing mild therapeutic HT with controlled NT after cardiac arrest. In a rat model of resuscitated cardiac arrest, Jia et al. [14] found increased electroencephalography (EEG) bursts, followed by earlier restoration of continuous EEG activity and lower neurological deficit in rats treated with mild therapeutic HT compared with NT or hyperthermic animals. In a recent retrospective chart review, Horburger et al. [13] described that therapeutic HT is associated with a significant improvement in neurological outcome and 180-day survival compared with spontaneous NT in out-of-hospital cardiac arrest patients.

These studies are consistent with the observations in the present study. We found that HT-treated animals had higher blood pressure and brain oxygen saturation with lower norepinephrine consumption. However, these differences reached statistical significance only during the final 30 min of reperfusion, after successful defibrillation in all hypothermic animals whereas hemodynamically effective heart rhythm was restored in only one of four normothermic pigs. Despite that the rates of successful defibrillation were not significantly different it is likely that the increased blood pressure and brain oxygenation in the hypothermic group were influenced by higher rate of successful resuscitation. Moreover, the blood pressure differences between the HT and NT animals could be further increased by the continuous ECMO support, irrespective of the defibrillation results. On the other hand, blood pressure, tissue saturation, vasopressor consumption and heart rhythm are dependent parameters; therefore, from a clinical point of view, it is difficult to speculate which of these factors plays a more important role. Of note, the numerical values of blood pressure in the only NT animal with the return of effective heart rhythm after defibrillation were at least 10 mmHg lower than the corresponding values from all hypothermic pigs despite higher norepinephrine dose.

We also observed lower levels of TnI, CPK, MBG and ALT in the HT group, indicating a protective effect of HT against myocardial, skeletal muscle and liver damage. The levels of CysC and NSE, markers of kidney and brain injury, were comparable in hypothermic and normothermic animals; however, these parameters were close to the lower limit of detection in both groups. It can, therefore, be assumed that 90 min of reperfusion is too short period to detect a substantial release of these markers into the circulation.

The lower levels of ROM during reperfusion in the HT group, together with comparable levels of BAP between groups, indicate that reactive oxygen species formation may be suppressed by HT, and a lower level of oxidative stress burden may occur in HT compared with NT. These results are consistent with our previous observations in the human population, in which induction of mild HT in cardiac arrest survivors was associated with a decreased production of nitrotyrosine and nitric oxide [15].

Our study has several limitations. First, we used only four animals per group that were followed for only ninety min after cardiac arrest; however, the present study was designed as a pilot experiment comparing the effects of HT and NT on clinically relevant parameters in a large-animal model. Second, the restoration of blood flow by ECMO in most cardiac arrest cases is not typically performed in clinical settings. However, ECMO was used to eliminate the individual variability of blood flow after resuscitation, and ensured ‘successful’ restoration of circulation in all animals. Finally, the measurement of CysC and NSE levels as markers of kidney and brain injury failed to be usable for early organ injury detection; other markers should be used in future studies of similar design.

## Conclusions

In porcine model of cardiac arrest, mild HT (33°C) was superior to controlled NT (36.8°C) for the maintenance of blood pressure and cerebral oxygenation, prevention of organ damage and oxidative stress suppression after cardiac arrest. Prospective clinical trials are needed to confirm these experimental results, some of which are already underway.

## Abbreviations

ALT: Alanin-aminotransferase; BAP: Biological antioxidant potential; CA: Cardiac arrest; CPC: Cerebral performance category; CPK: Creatine-phosphokinase; CysC: Cystatin C; ECMO: Extracorporeal membrane oxygenation; EEG: Electroencephalography; HT: Mild hypothermia; MGB: Myoglobin; NSE: Neuron-specific enolase; NT: Controlled normothermia; ROM: Reactive oxygen metabolite; TnI: Troponin I.

## Competing interests

The authors declare that they have no competing interests.

## Authors’ contributions

PO, MM, OK, PN: study conception and design; AK, SH, MS, FH, TS, VH: animal experiment conduction, hemodynamic data acquisition, analysis, and interpretation; AK, HP: oxidative stress data acquisition, analysis, and interpretation; LT carried out immunoassays; VD carried out immunoradiometric assays; PO: manuscript drafting; JB, OK, PN: critical revision and final approval of the manuscript. All authors have read and approved the manuscript for publication.
